# Response to the Letter to the Editor

**DOI:** 10.1111/anec.12720

**Published:** 2019-10-15

**Authors:** Nobuhiro Takasugi, Hiroko Matsuno, Mieko Takasugi, Koichi Shinoda, Takatomo Watanabe, Hiroyasu Ito, Hiroyuki Okura, Richard L. Verrier

**Affiliations:** ^1^ Gifu University Hospital Gifu Japan; ^2^ Matsunami General Hospital Gifu Japan; ^3^ Harvard Medical School Beth Israel Deaconess Medical Center Boston MA USA

Dear Editor

We read Dr. Selvaraj's letter with interest and appreciate his intention to enhance the accuracy of the modified moving average (MMA) method for detection of microvolt levels of T‐wave alternans (TWA). Unfortunately, the methodology that he and his colleague, Dr. Chauhan, developed and evaluated (Selvaraj & Chauhan, 2009) is based on their custom software, which differs in many important respects from the MMA software that was FDA‐cleared for risk stratification for arrhythmic death (FDA document K032513), commercialized by GE Healthcare, and used multinationally by independent investigators in studies that enrolled over 6,000 patients (Verrier et al., 2011).

Dr. Selvaraj's TWA software, which is not FDA‐cleared or shown to stratify risk, does not include extensive preprocessing and noise‐reduction algorithms developed over many years of commercial ECG monitoring and uses a 1/16 rather than 1/8 update factor, which impacts on TWA measurement. Also, his version of the MMA algorithms does not use templates of QRS‐aligned superimposed median beat complexes, which allow automatically generated high‐resolution morphology complex display and visual verification of the TWA level down to 20 µV, well below the 47‐ and 60‐µV cutpoints of abnormality and severe abnormality (Verrier et al., 2011). This crucial omission from their version of MMA software leaves out a critical feature that was utilized in the current study (Takasugi et al., 2019) to enhance the precision of the MMA method.

We identified three main sources of potential measurement error (Takasugi et al., 2019), namely artifacts primarily associated with limb leads, T‐wave changes due to heart and body position observed mainly in the right precordial leads V_1_ and V_2_, and postextrasystolic T‐wave changes, which can be readily identified. By addressing these potential sources of measurement error, which can readily be performed using the automated median beat templates, the accuracy of MMA analysis can be further improved. Effects of respiration were removed by commercial filters; thus, our work (Takasugi et al., 2019) should not be invoked to corroborate the findings of Drs. Selvaraj and Chauhan (Selvaraj & Chauhan, 2009), given the fundamental differences in methodology.

Finally, we do not believe that Dr. Selvaraj's suggestion of ratioing alternans to noise is suitable as it eliminates the potential to quantify TWA level, which has been shown to reflect the degree of risk and to indicate the impact of medical therapy (Verrier et al., 2011). In fact, risk of cardiovascular mortality and risk of sudden cardiac death increase by 55% and 58%, respectively, per 20 µV of TWA (Verrier et al., 2011).

It would be valuable if Dr. Selvaraj chooses to evaluate further the MMA approach that he use the FDA‐cleared algorithms, which are in current clinical use.
